# Heterologous expression of pikromycin biosynthetic gene cluster using *Streptomyces* artificial chromosome system

**DOI:** 10.1186/s12934-017-0708-7

**Published:** 2017-05-31

**Authors:** Hye-rim Pyeon, Hee-Ju Nah, Seung-Hoon Kang, Si-Sun Choi, Eung-Soo Kim

**Affiliations:** Department of Biological Engineering, Inha University, Incheon, 402-751 South Korea

**Keywords:** *Streptomyces* artificial chromosome, Pikromycin biosynthetic gene cluster, Heterologous expression

## Abstract

**Background:**

Heterologous expression of biosynthetic gene clusters of natural microbial products has become an essential strategy for titer improvement and pathway engineering of various potentially-valuable natural products. A *Streptomyces* artificial chromosomal conjugation vector, pSBAC, was previously successfully applied for precise cloning and tandem integration of a large polyketide tautomycetin (TMC) biosynthetic gene cluster (Nah et al. in Microb Cell Fact 14(1):1, 2015), implying that this strategy could be employed to develop a custom overexpression scheme of natural product pathway clusters present in actinomycetes.

**Results:**

To validate the pSBAC system as a generally-applicable heterologous overexpression system for a large-sized polyketide biosynthetic gene cluster in *Streptomyces*, another model polyketide compound, the pikromycin biosynthetic gene cluster, was preciously cloned and heterologously expressed using the pSBAC system. A unique *Hind*III restriction site was precisely inserted at one of the border regions of the pikromycin biosynthetic gene cluster within the chromosome of *Streptomyces venezuelae*, followed by site-specific recombination of pSBAC into the flanking region of the pikromycin gene cluster. Unlike the previous cloning process, one *Hind*III site integration step was skipped through pSBAC modification. pPik001, a pSBAC containing the pikromycin biosynthetic gene cluster, was directly introduced into two heterologous hosts, *Streptomyces lividans* and *Streptomyces coelicolor,* resulting in the production of 10-deoxymethynolide, a major pikromycin derivative. When two entire pikromycin biosynthetic gene clusters were tandemly introduced into the *S. lividans* chromosome, overproduction of 10-deoxymethynolide and the presence of pikromycin, which was previously not detected, were both confirmed. Moreover, comparative qRT-PCR results confirmed that the transcription of pikromycin biosynthetic genes was significantly upregulated in *S. lividans* containing tandem clusters of pikromycin biosynthetic gene clusters.

**Conclusions:**

The 60 kb pikromycin biosynthetic gene cluster was isolated in a single integration pSBAC vector. Introduction of the pikromycin biosynthetic gene cluster into the pikromycin non-producing strains resulted in higher pikromycin production. The utility of the pSBAC system as a precise cloning tool for large-sized biosynthetic gene clusters was verified through heterologous expression of the pikromycin biosynthetic gene cluster. Moreover, this pSBAC-driven heterologous expression strategy was confirmed to be an ideal approach for production of low and inconsistent natural products such as pikromycin in *S. venezuelae*, implying that this strategy could be employed for development of a custom overexpression scheme of natural product biosynthetic gene clusters in actinomycetes.

**Electronic supplementary material:**

The online version of this article (doi:10.1186/s12934-017-0708-7) contains supplementary material, which is available to authorized users.

## Background

Microbial natural products including secondary metabolites produced by actinomycetes have been a major resource for new drug discovery and development because of their superior structural diversity and complexity [1]. Although identification of entire biosynthetic gene clusters has become relatively straightforward because of genome mining and next generation sequencing, some of the biosynthetic genes are derived from non-culturable organisms or from microorganisms that are not amenable to genetic manipulation and are therefore not easily expressed for target compound identification [2]. To overcome such intrinsic limitations and achieve functional expression of uncharacterized potentially-valuable natural product biosynthetic pathways, relatively well-characterized heterologous host expression strategies have been pursued.

The cosmid/fosmid library system has been used extensively for several decades to enable heterologous expression of natural product biosynthetic gene clusters of actinomycetes [3–8]. Recently, several sophisticated heterologous expression approaches have been introduced, including the linear plus linear homologous recombination (LLHR) system [9], transformation-associated recombination (TAR) system [10] and *Streptomyces* bacterial artificial chromosome (pSBAC) system [11]. Specifically, Yamanaka et al. designed the TAR cloning vector, pCAP01, which consists of a yeast element, an *Escherichia coli* element, and an actinobacterial element. The marinopyrrole biosynthetic gene cluster (30 kb) and taromycin A biosynthetic gene cluster (67 kb) were captured by a TAR system using yeast recombination activity, then functionally expressed in *Streptomyces coelicolor* [10]. Although a TAR system might be suitable for cloning and expression of a large-sized cryptic gene cluster screened from actinomycetes genome mining, TAR cloning must be conducted in yeast before intergeneric conjugation into *Streptomyces*. Additionally, Yin et al. [12] cloned a large-sized salinomycin biosynthetic gene cluster by linear plus linear homologous recombination (LLHR) using the Red/ET system and successfully expressed it in *S. coelicolor*. However, because the efficiency of this system is low for a large cluster (>60 kb), the cluster should be divided for serial assembly. In addition, both TAR and LLHR systems were mediated by homologous recombination, so undesired recombination could appear.

We previously successfully demonstrated that the pSBAC system is as an efficient heterologous expression system with site-specific restriction site insertion, recombinant pSBAC plasmid rescue, and *E. coli*–*Streptomyces* intergeneric conjugation. Using this pSBAC system, we isolated a large-sized TMC biosynthetic gene cluster (80 kb) from *Streptomyces* sp. CK4412 and expressed it in both homologous and heterologous hosts [13]. Since the pSBAC cloning approach uses the plasmid rescue method, undesired recombination does not appear and overexpression of the target gene cluster is possible through simple antibiotic marker substitution.

In this study, another large-sized polyketide pikromycin biosynthetic gene cluster, which was discovered in early 1950s and examined by many researchers for identification of the mechanism of polyketide elongation and their structural changes [14–16], was cloned and directly expressed in two different heterologous hosts using the pSBAC system. Moreover, tandem integration of the pikromycin cluster-containing pSBAC in *Streptomyces lividans* resulted in significantly enhanced productivities of both 10-deoxymethynolide and pikromycin, implying that this pSBAC system might be an efficient strategy for functional overexpression of the entire biosynthetic gene cluster of any potentially-valuable low-titer metabolite in actinomycetes.

## Results

### Isolation of pikromycin biosynthetic gene cluster using the pSBAC system

**Fig. 1:**
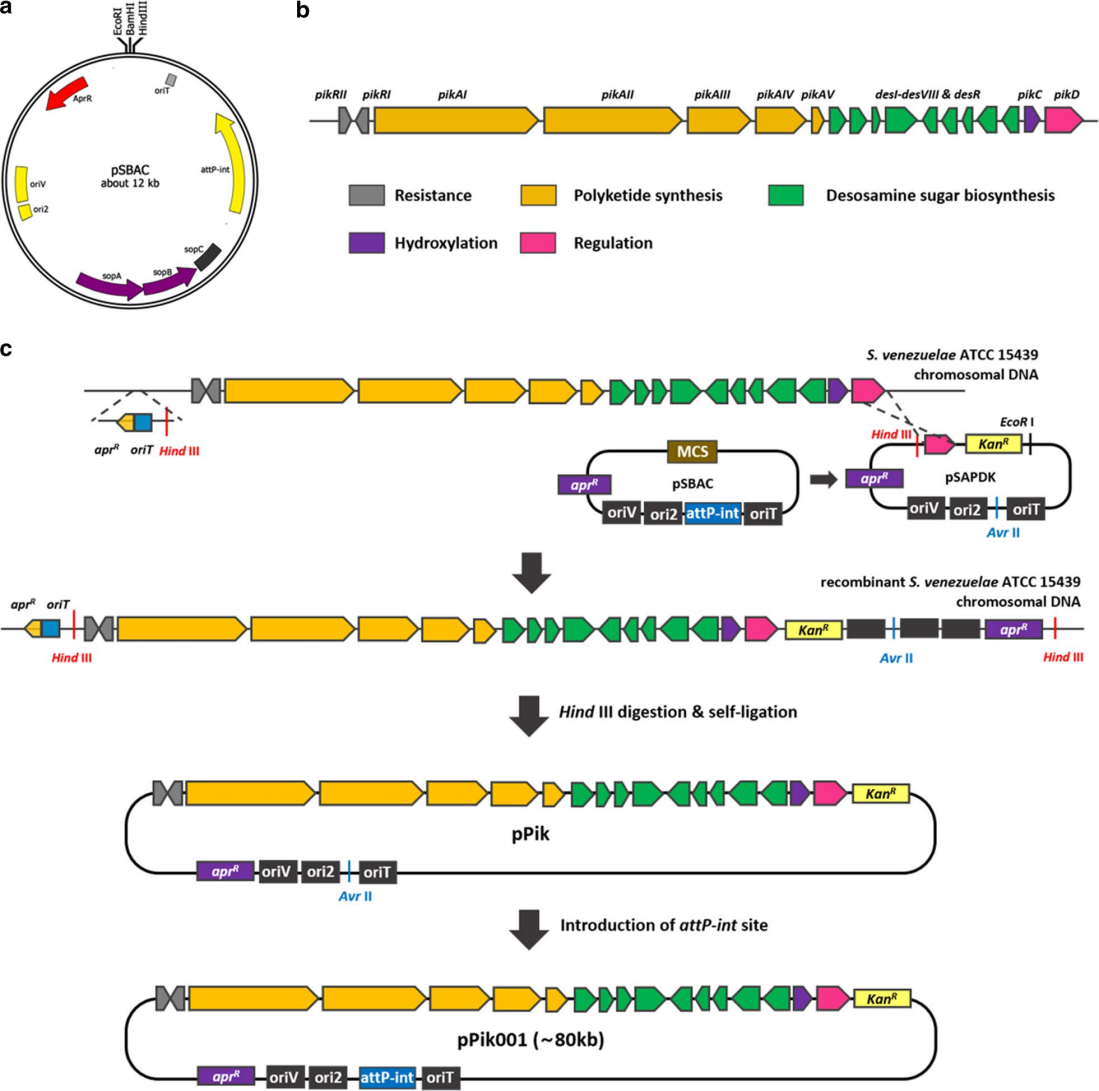
**a** Map of pSBAC. Essential components of the vector are indicated. *Ori2* and *oriV*, replication origins; *sopA*-*C*, partitioning system; *apr* ^*R*^, apramycin resistance gene; *oriT*, origin of transfer; *attP*-*int*_ΦBT1_, integration system; unique restriction enzyme recognition sites, *Eco*RI, *Bam*HI and *Hind*III. **b** Composition of pikromycin biosynthetic gene cluster. *pikAI*-*V*, polyketide synthesis; *desI*-*VIII,* desosamine sugar synthesis; *pikC,* hydroxylation; *pikD,* regulation; *pikRI*-*II,* resistance. **c** Schematic description of pPik001 construction. One of *Hind*III recognition sites was introduced into the 150 bp upstream of *pik*RII and another *Hind*III recognition site was integrated next to *pikD* in pSAPDK. The pikromycin biosynthetic gene cluster was isolated by *Hind*III digestion of the modified chromosomal DNA and its self-ligation, then named pPik. After construction of pPik, a DNA fragment containing *attP*-*int*_ΦBT1_ was inserted into the *Avr*II recognition site of pPik to generate pPik001

There were only two examples of pSBAC-driven heterologous expression systems in *Streptomyces*, meridamycin (95 kb) and TMC (80 kb) biosynthetic gene clusters [11, 13]. While the entire meridamycin gene cluster was captured in a single pSBAC clone via straightforward restriction enzyme digestion because of the presence of unique restriction enzyme *Mfe*I sites in border regions of the meridamycin biosynthetic gene cluster, unique *Xba*I restriction sites were precisely inserted at both border regions of the TMC biosynthetic gene cluster within the chromosome of *Streptomyces* sp. CK4412. Since the pikromycin biosynthetic gene cluster does not possess a unique restriction enzyme site in the borders like the TMC gene cluster, a unique restriction enzyme site was first inserted at the borders of the pikromycin gene cluster on the *S. venezuelae* ATCC 15439 chromosome. At one side of the pikromycin biosynthetic gene cluster near *pikRII*, a *Hind*III restriction enzyme site was inserted into 150 bp upstream region from *pikRII* using PCR targeted gene insertion [17]. To accomplish this, pMSCpik311 containing an apramycin resistance gene, *oriT* and a *Hind*III restriction enzyme site was constructed in *E. coli* based on pSCpik311, the pikromycin border-containing cosmid. The modified cosmid was introduced into *S. venezuelae* ATCC 15439 by conjugation, followed by target sequence-specific recombination at the border of the pikromycin gene cluster (Fig. 1). The resulting ex-conjugants were selected with the antibiotic selection marker, and insertion of *Hind*III in the correct site was confirmed by PCR analysis and sequencing (Additional file 1: Figure S1). For the other side of the cluster, a *Hind*III restriction enzyme site in the multiple cloning site of pSBAC was used as a restriction enzyme site for plasmid rescue method in the vicinity of *pikD*. To integrate a pSBAC vector into the recombinant *S. venezuelae* ATCC 15439 chromosome, a gene cassette containing a segment of *pikD* and the kanamycin resistance gene was cloned into a pSA (pSBAC Δ*attP*-*int*_ΦBT1_) using the *Hind*III and *Eco*RI restriction enzyme sites. The resulting construct was named pSAPDK, then directly introduced into *S. venezuelae* ATCC15439. pSAPDK vector was integrated into the border near *pikD* through homologous recombination, and integration of pSAPDK was confirmed by PCR analysis (Additional file 1: Figure S1). *Hind*III-digested total chromosomal DNA fractions were self-ligated, after which they were transformed into *E*. *coli* cells. Transformed strains were selected using apramycin and kanamycin antibiotics and then confirmed by PCR, enzyme mapping, and sequencing (Additional file 2: Figure S2). Finally, the DNA fragment containing *attP*-*int*_ФBT1_ was re-introduced into the rescued recombinant pSBAC vector and named pPik001 (Fig. 1).

### Heterologous expression of pikromycin biosynthetic gene cluster in *Streptomyces* strains

**Fig. 2:**
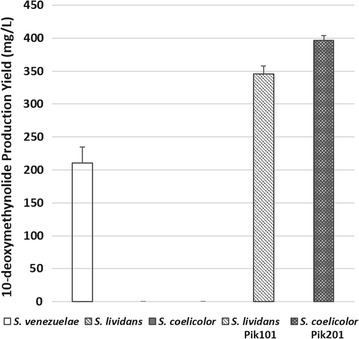
Comparison of 10-deoxymethynolide production in *S. venezuelae, S. lividans* wild type*, S. lividans* Pik101, *S. coelicolor* wild type and *S. coelicolor* Pik201

**Fig. 3:**
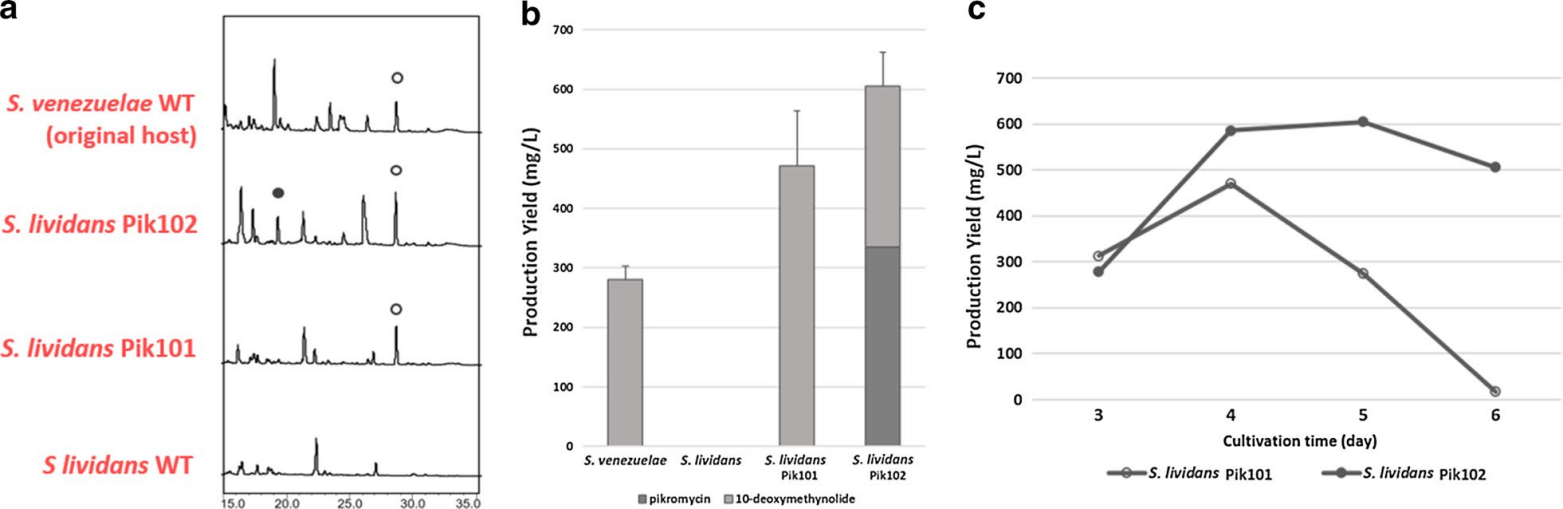
**a** HPLC profiles of *S. venezuelae*, *S. lividans* Pik102, *S. lividans* Pik101 and *S. lividans*: *open circle*, 10-deoxymethynolide; *closed circle*, pikromycin. **b** The production yield of pikromycin and 10-deoxymethynolide in *S. venezuelae, S. lividans* Pik101 and *S. lividans* Pik102. **c** The production yield of pikromycin and 10-deoxymethynolide in *S. lividans* Pik101 and *S. lividans* Pik102 for 4 days; *open circle*, *S. lividans* Pik101; *closed circle*, *S. lividans* Pik102

To confirm expression of the pikromycin biosynthetic gene cluster in pPik001, it was introduced into both *S. lividans* and *S. coelicolor* strains through conjugation, which generated *S. lividans* Pik101 and *S. coelicolor* Pik201, respectively. The constructed strains were cultured along with wild-type *S. venezuelae* ATCC 15439. Although pikromycin was not detected from wild-type or heterologous hosts, highly noticeable peaks showing identical retention times and spectra were detected in all three cultures (Fig. 3a, data not shown for *S. coelicolor* Pik201). The collected fraction of the peak was analyzed with high resolution LC/MS and confirmed to be 10-deoxymethynolide, a major pikromycin derivative released from module 5 in pikromycin biosynthetic gene cluster. The results of LC/MS also confirmed expression of the pikromycin biosynthetic gene cluster and that the yields of 10-deoxymethynolide in *S. lividans* Pik101 and *S. coelicolor* Pik201 were 346.8 and 396.2 mg/L, respectively. When the production yield of the heterologous hosts was compared with the wild-type, *S. lividans* Pik101 and *S. coelicolor* Pik201 exhibited 1.6-fold and 1.8-fold increases, respectively (Fig. 2), revealing that the pSBAC-driven heterologous expression of an entire pikromycin biosynthetic gene cluster resulted in enhanced production in both *S. lividans* and *S. coelicolor*.

### Heterologous tandem integration of entire pikromycin cluster

**Fig. 4:**
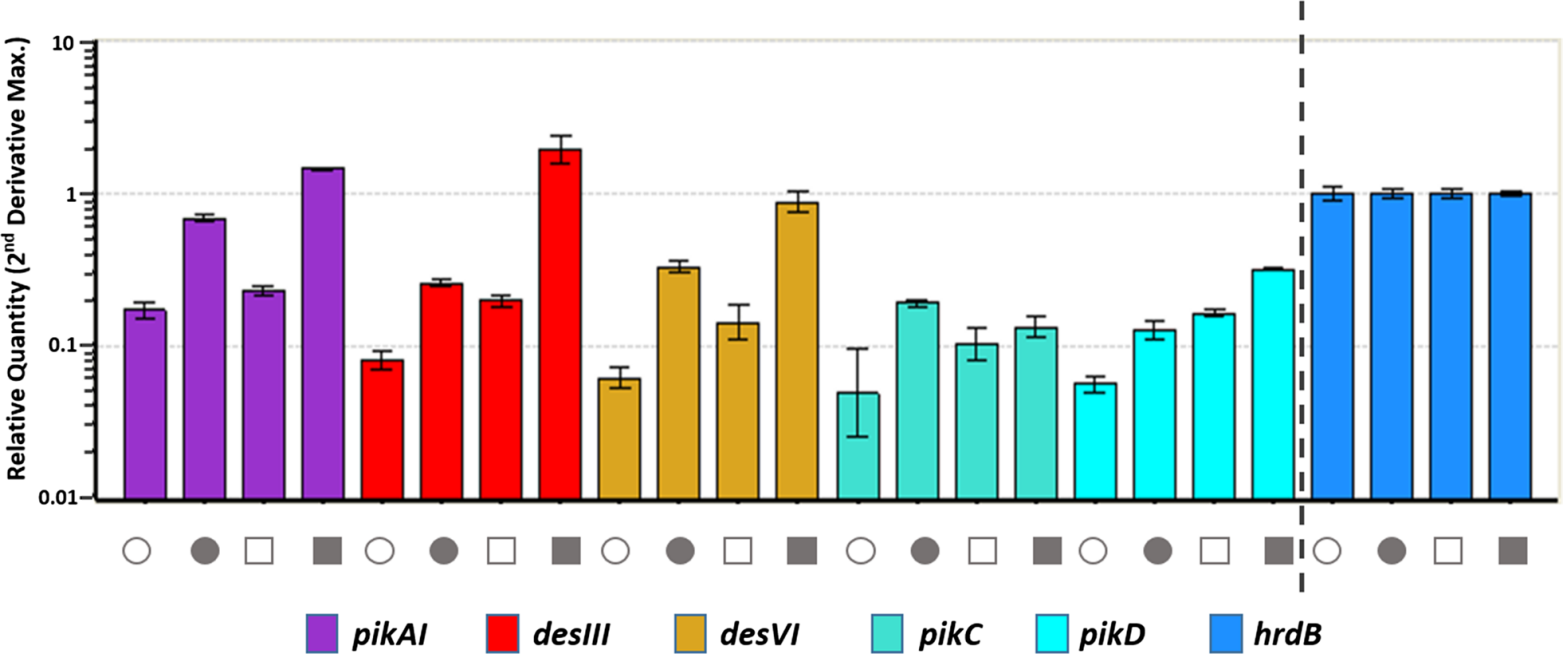
Transcript analysis of pikromycin gene cluster expression. *Circle*, sample was taken on day 3; *square*, sample was taken on day 5; *open circle* and *square*, *S. lividans* Pik101; *closed circle* and *square*, *S. lividans* Pik102

To further stimulate pikromycin productivity, an additional copy of the pikromycin cluster was introduced into *S. lividans* Pik101 containing a single-copy of pikromycin. To introduce the second copy of the pikromycin gene cluster, the apramycin resistance gene in pPik001 was substituted with a hygromycin resistance gene and named pPik003. Next, pPik003 was introduced into *S. lividans* Pik101 through conjugation, after which the exconjugant was selected using both hygromycin and apramycin. Four exconjugants were randomly chosen to confirm the tandem integration by PCR amplification of attP-int primer sets. In all four selected colonies, the pPik003 was integrated into adjacent region of the pPik001 by homologous recombination. The resulting strain was named *S. lividans* Pik102. Although pikromycin was not detected in both *S. venezuelae* and *S. lividans* Pik101 as stated above, pikromycin production was observed in *S. lividans* Pik102 with the pikromycin yield of 333.7 mg/L (Fig. 3a). Moreover, the total production of pikromycin and 10-deoxymethynolide in *S. lividans* Pik102 (604.7 mg/L) showed a 2.1-fold increase compared to the production of only 10-deoxymethynolide in the parental wild-type strain (280 mg/L) (Fig. 3b). Additionally, the yield of *S. lividans* Pik102 was 1.2-fold higher than that of *S. lividans* Pik101. Production of the compounds in *S. lividans* Pik102 remained high until day 6 of culture, whereas production in *S. lividans* Pik101 gradually decreased from day 4 of culture (Fig. 3c). Comparative qRT-PCR results also confirmed that transcription of four biosynthetic genes (*pikAI, pikC, desIII and desVI*), as well as the pathway-specific regulatory gene *pikD*, was significantly stimulated in *S. lividans* Pik102 (Fig. 4). These findings imply that the presence of an additional copy of the entire pikromycin biosynthetic gene cluster was responsible for the increased transcription of pikromycin biosynthetic genes.

## Discussion

The *E. coli*–*Streptomyces* shuttle vector, pSBAC, is an efficient tool that is capable of accepting and stably maintaining a large-sized natural product biosynthetic gene cluster [11]. Although we previously demonstrated an efficient strategy for giant plasmid rescue and tandem overexpression of a large-sized TMC biosynthetic gene cluster both in homologous and heterologous hosts [13], another example of a natural product biosynthetic gene cluster with different restriction enzyme site cloning was needed to be accepted as a general heterologous expression system in *Streptomyces*. Therefore, a pikromycin biosynthetic gene cluster was isolated using the pSBAC system as another example. The pikromycin biosynthetic gene cluster has some problems in that diverse derivatives were seen in a production culture, production varied in flasks and pikromycin production yield was quite low, making it difficult to detect. Because the pikromycin biosynthetic gene cluster was expressed in heterologous hosts, the production yield of 10-deoxymethynolide, one of pikromycin derivatives, was 1.6- and 1.8-fold of that of the parental wild-type host in *S. lividans* Pik101 and *S. coelicolor* Pik201, respectively. Moreover, pikromycin was detected in *S. lividans* Pik102, which has two copies of pikromycin biosynthetic gene cluster. These results suggest that a bottleneck exists in the pikromycin biosynthetic pathway that could be solved by introducing additional pikromycin biosynthetic genes. Expression of the pikromycin biosynthetic gene cluster was also confirmed through real-time RT-PCR at the transcriptional level. As expected, the level of transcripts of pikromycin biosynthetic genes in *S. lividans* Pik102 was higher than that in *S. lividans* Pik101 on both day 3 and 5. Although a significant gap in expression levels between *S. lividans* Pik101 and *S. lividans* Pik102 was shown, the compound yield in *S. lividans* Pik102 did not differ significantly from that of *S. lividans* Pik101. These findings suggest that the pikromycin gene cluster was amply transcribed, but that another bottleneck may exist in pikromycin production, such as a shortage of building blocks. In conclusion, the *Streptomyces* pSBAC-driven precise cloning and tandem integration approach described here can be applied to the versatile overexpression of large secondary metabolite gene clusters in actinomycetes, including novel cryptic clusters identified by genome mining.

## Conclusions

Although actinomycetes have provided abundant sources for the development of new pharmaceuticals, their widespread application for this purpose is hindered by low productivity and difficult cultivation. Because many cryptic pathways have been found through genome mining, heterologous expression was suggested as a useful method for production of new pharmaceuticals. Development of an essential tool that can isolate, maintain, and express a large-sized gene cluster has emerged as a major point of interest. We previously demonstrated that the pSBAC system was an attractive genetic system for the efficient heterologous overexpression of a target cluster in *Streptomyces* species. However, the process for isolation of a biosynthetic gene cluster still requires a series of conjugation and selection steps. Here, we isolated the pikromycin biosynthetic gene cluster as a model pathway using the pSBAC system and simplified the process by eliminating one conjugation step using a *Hind*III restriction enzyme site in multiple cloning sites of pSBAC for plasmid rescue. Introduction of two copies of the pikromycin biosynthetic gene cluster into *S. lividans* was confirmed to stimulate the production of both 10-deoxymethynolide and pikromycin. Overall, the results of this study showed natural microbial products that are usually not produced or produced in very low amounts could be generated via overexpression of a biosynthetic gene cluster. This study confirms that the pSBAC system is a generally-applicable technology for the precise cloning and functional overexpression of the large-sized biosynthetic gene cluster of any potentially-valuable low-titer metabolites in actinomycetes.

## Methods

### Bacterial strains and culture media

The bacterial strains and plasmids used in this study were summarized in Table 1. *E. coli* strains were cultured in Luria–Bertani (LB) broth or on Luria–Bertani agar supplemented with appropriate antibiotics at 37 °C. For production of pikromycin and its derivatives, the original host, *S. venezuelae,* was grown in SGGP media for 1 day, then cultured for 3 days in SCM media at 30 °C [15]. Additionally, the model strains *S. lividans* and *S. coelicolor* were grown in TSB media for 2 days, then cultured for 5 days in R5 media at 30 °C [18]. Conjugation was carried out on modified ISP4 medium.

**Table 1:** Bacterial strains and plasmids used in this study

Strain/plasmid	Relevant characteristics	Source/references
Plasmid		
pSBAC	*aacIII*(IV), *oriT*, *attP*-*int*_ΦBT1_, backbone of pCC1BAC	[11]
pSAPDK	Modified pSBAC with deleted *attP*-*int*_ΦBT1_ and inserted *Kan* ^*R*^ and *pikD* fragment	This work
pPik	pSAPDK with 60 kb DNA insert containing whole *pik* gene cluster	This work
pPik001	pPik with *attP*-*int*_ΦBT1_	This work
pPik003	pPik101, which replaced *Hyg* ^*R*^ with *Apr* ^*R*^	This work
*E. coli*		
EPI300	F-*mcrA*-*D* (*mrr*-*hsd*RMS-*mcrBC*) *trfA* host for cloning and amplification of various BAC vectors and constructs derived from it	Epicenter
S17-1	*E. coli* host for transferring various plasmids into *Streptomyces* via conjugation	
ET12567/pUZ8002	*E. coli* host for transferring various plasmids into *Streptomyces* via conjugation	
*Streptomyces venezuelae*		
ATCC 15439	Original pikromycin-producing strain	[15]
15439-HindIIIBAC	ATCC 15439 with pSAPDK and *Hind*III recognition sequences in a flanking region of pikromycin biosynthetic gene cluster	This work
*Streptomyces lividans*		
TK21	Non pikromycin-producing strain	
Pik101	TK21 with pPik001	This work
Pik102	Pik101 with pPik003	This work
*Streptomyces coelicolor*		
M145	Non pikromycin-producing strain	
Pik201	M145 with pPik001	This work

### Insertion of unique *Hind*III recognition sequence into a flanking region of the pikromycin biosynthetic gene cluster

To isolate the pikromycin biosynthetic gene cluster, a unique *Hind*III recognition sequence was inserted into a flanking region of the pikromycin biosynthetic gene cluster using a PCR-targeted gene disruption system [17]. An apramycin resistance gene *(aac(3)IV)/oriT* cassette was used to insert a *Hind*III recognition sequence into a flanking region near *pikRII*. This cassette was amplified from pIJ773 using the primers containing the *Hind*III recognition sequence and a homologous sequence with a flanking region near *pikRII* at the 5′- and 3′-ends, respectively. The cassette was then introduced into *E. coli* BW25113/pIJ790 containing pSCPik311, resulting in pSCPik311*::aac(3)IV/oriT*, pMSCPik311. Insertion of the *Hind*III recognition sequence was confirmed by PCR amplification and sequencing (Macrogen, Korea). The mutated cosmid, pMSCPik311, was subsequently introduced into *E. coli* ET12567/pUZ8002 and then was directly conjugated with *S. venezuelae* ATCC 15439. Conjugation experiments were then performed as previously described [11].

### Isolation of entire pikromycin biosynthetic gene cluster into pSBAC

To isolate the entire pikromycin biosynthetic gene cluster from the chromosome by *Hind*III digestion and ligation, *attP*-*int*_ΦBT1_ was removed from pSBAC by *Avr*II digestion and self-ligation, and the modified vector was named pSA. To integrate pSA into the vicinity of *pikD* by homologous recombination and select the correct colonies, a cassette containing a 1879-bp DNA fragment including a portion of *pikD* and a kanamycin resistance gene was constructed by 3-way PCR amplification using primers containing the restriction enzyme sites, *Hind*III and *Eco*RI, respectively. The amplified PCR product was ligated into a T&A cloning vector (RBC), after which the ligated vector was sequenced to confirm its integrity (Macrogen, Korea). The cassette was then digested using *Hind*III and *Eco*RI, after which it was cloned into *Eco*RI–*Hind*III-digested pSA to generate pSAPDK. Conjugation was subsequently performed to integrate pSAPDK into the chromosomal DNA in *S. venezuelae* via homologous recombination. Next, the desired mutants were selected on apramycin- and kanamycin-containing ISP4 agar, verified using PCR and named *S. venezuelae* HindBAC. This strain was cultured in TSB media for 1 day at 30 °C, after which its genomic DNA was prepared using a Wizard^®^ genomic DNA purification kit (Promega). Genomic DNA was then digested by restriction enzyme *Hind*III, purified, and concentrated by ethanol precipitation before self-ligation using T4 ligase (TaKaRa). After desalting, the ligation mixture was used for electroporation of *E. coli* EPI300. Recombinants were selected on apramycin- and kanamycin-containing LB medium, after which plasmids were isolated by alkali denaturation and screened by PCR using PikRI-AI, PikAII, PikD and PikD-kan check primers in pikromycin cluster to identify pPik. The *attP*-*int*_ΦBT1_ was digested by *Avr*II from the *attP*-*int*_ΦBT1_-containing vector constructed in a previous study and then cloned into pPik to generate pPik001.

### Extraction and HPLC quantification of 10-deoxymethynolide

Each 50 mL culture medium was centrifuged at 6000×*g* for 15 min. Pikromycin and its derivatives were extracted from the supernatant with two volumes of ethyl acetate and concentrated using a rotary evaporator, after which the final extracts were dissolved in methanol. Analytical HPLC analysis was conducted using an Agilent C18 5-μm column with 80% acetonitrile in 5 mM ammonium acetate buffer containing 0.05% acetic acid at a flow rate of 1 mL/min. Detection was performed at 220 nm [19].

### Replacement of apramycin-resistant gene by other antibiotic resistance genes and introduction into *pik*-containing strain

To introduce the additional pikromycin cluster into *S. lividans* Pik101, the apramycin-resistant gene of pPik001 was replaced by a hygromycin-resistant gene using a Quick and Easy BAC modification kit (GeneBridges), after which it was denoted pPik003. Next, pPik001 was introduced into Red/ET plasmid-containing *E. coli* EPI300, after which BAC modification was performed in accordance with the manufacturer’s guide using the hygromycin-resistant gene containing the *apr* ^*R*^-homologous arm constructed by PCR. Transformants were selected on hygromycin containing LB medium and confirmed by PCR. After pPik003 was transformed into *E. coli* S17-1, it was introduced into *S. lividans* Pik101 by conjugation.

### Isolation of total RNA and gene expression analysis by qRT-PCR

For RNA preparation, *S. lividans* TK21, *S. lividans* Pik101 and *S. lividans* Pik102 were grown for 6 days in R5 medium, during which time samples were collected on day 3 and 5. Mycelia were harvested by centrifugation and stored in a −80 °C deep freezer after washing twice with distilled water. RNA preparation and qRT-PCR were then carried out as previously reported [20] using the primers shown in Table 2.

**Table 2:** Primer list used in this study

Oligonucleotide	Primer sequence (5′–3′)	Purpose
Homo arm-apr^R^ cassette		
F	TACCCGGCGGAGGCGTACCACCAGCAGTATCTGGACAAGAAATTCCGGGGATCCGTCGACC	Construction of pMSCPik311
R	AGCGGCGCAGCCTCATGTAACGGGCCTGAGAGTGGCAGAAGCTTGCATTGTAGGCTGGAGCTGCCTC	
Apr^R^ cassette check		
F	TACGGCGAGATCACCACGAC	Check for insertion of *apr* ^*R*^ cassette
R	GCGAGGATCGAGACGCGTGA	
PikD-kan		
F	ACAACACCAAGCTTGCCCGCCT	Construction of pSAPDK
R	ACTGCAAGCTAGGCCCACCGATGAAGGACA	
F	TCGGTGGGCCTTAGCTTGCAGTGGGCTTACA	
R	TTGGTCGGTGAATTCGAACCCCAGA	
PikD-kan check		
F	AGGCCCTGTTCGACGACCGA	Confirmation of pPik001
R	TGCCTCGTCCTGCAGTTCATTCA	
PikRI-AI		
F	CCCCTTCGAATTTCCGCCGC	Confirmation of pPik001
R	CCGCCAGGAGTTCCCAGAAC	
PikAII		
F	ACCGGTCTCAACTTCCGCGA	Confirmation of pPik001
R	TCCAGGAGGGACAGGGTCAGC	
PikD		
F	TCGCGGCACGGCATGGAGTACATGCA	Confirmation of pPik001
R	TCAGGCCGTGACGGACTTGTCCT	
PikAI RNA		
F	GACTGGGACCGCTTCTACCT	qRT-PCR
R	TACGAGACCGAGGAGGATCT	
DesIII RNA		
F	GCTCTTTCTGGACCGCATAG	qRT-PCR
R	CGGTCATTTCGAAGCAGATT	
DesVI RNA		
F	CATCTGGAGCACTTCACCAA	qRT-PCR
R	CTCGGTCGTCTTCAGGTAGC	
PikC RNA		
F	GCTATCTCTCCCGGCTCATC	qRT-PCR
R	GATTGACCGTGGTCTCGTG	
PikD RNA		
F	GGACGCTCTTACTCGTCTCC	qRT-PCR
R	CGGAGTAACTGGCAGAGGAC	
HrdB RNA		
F	GCGGTGGAGAAGTTCGACTA	qRT-PCR
R	CACATGGTCGAGGTCATCAA	

## Additional file

**Additional file 1: Figure S1.** (A) Confirmation of *Hind*III insertion near *pikRII* with *apr* ^*R*^ check primers shown in Table 2. The expected amplicon size of mutant and wildtype is 1.5kb and 360bp, respectively.; lane 1, 100 bp loading DNA ladder (DYNEBIO Inc.); lane 2 and 4, PCR products from *S. venezuelae* tDNA; lane 3 and 5, PCR products from *S. venezuelae* Hindbac tDNA; lane 4 and 5, *Hind*III digested PCR products. (B) Confirmation of integration of pSAPDK in the vicinity of *pikD*; lane 1, 1 kb DNA ladder (cosmogenetech); lane 2, PCR product from *S. venezuelae* Hindbac tDNA; lane 3, PCR product from *S. venezuelae* tDNA.

**Additional file 2: Figure S2.** Confirmation of pPik001 (A) PCR analysis using randomly selected primer within pikromycin biosynthetic gene cluster. Amplicons were loaded on a gel in order of pikD-kan (lane 2-3), PikAII (lane 4-5), pikRI-pikAI (lane 6-7), pikD check primers (lane 8-9). Lane 1, 1kb DNA ladder (Cosmo genetech); lane2, 4, 6, 8, *S. venezuelae* tDNA; lane 3,5,7,9, pPik001. (B) Enzyme mapping using various restriction enzymes. Digestion mixtures were loaded on a gel in order of *Bgl*II digestion (1-2), *Pst*I digestion (3-4), *Bgl*II and *Pst*I digestion (5-6). M, λ-*Hind*III DNA ladder; 1, 3, 5, pSBAC; 2, 4, 6, pPik001.
