# Supplementary material for: Heterologous expression of pikromycin biosynthetic gene cluster using Streptomyces artificial chromosome system
Source: Microb Cell Fact. 2017 May 31;16:96. doi: 10.1186/s12934-017-0708-7 (PMC5452415; doi:10.1186/s12934-017-0708-7)
Supplement: Supplementary file 1 — Additional file 1: Figure S1. (A) Confirmation of HindIII insertion near pikRII with apr R check primers shown in Table 2. The expected amplicon size of mutant and wildtype is 1.5kb and 360bp, respectively.; lane 1, 100 bp loading DNA ladder (DYNEBIO Inc.); lane 2 and 4, PCR products from S. venezuelae tDNA; lane 3 and 5, PCR products from S. venezuelae Hindbac tDNA; lane 4 and 5, HindIII digested PCR products. (B) Confirmation of integration of pSAPDK in the vicinity of pikD; lane 1, 1 kb DNA ladder (cosmogenetech); lane 2, PCR product from S. venezuelae Hindbac tDNA; lane 3, PCR product from S. venezuelae tDNA. [file 12934_2017_708_MOESM1_ESM.docx]

**Figure S1.** (A) Confirmation of *Hind*III insertion near *pikRII* with *apr^R^* check primers shown in Table 2*.* The expected amplicon size of mutant and wildtype is 1.5kb and 360bp, respectively.; lane 1, 100 bp loading DNA ladder (DYNEBIO Inc.); lane 2 and 4, PCR products from *S. venezuelae* tDNA; lane 3 and 5, PCR products from *S. venezuelae* Hindbac tDNA; lane 4 and 5, *Hind*III digested PCR products. (B) Confirmation of integration of pSAPDK in the vicinity of *pikD*; lane 1, 1 kb DNA ladder (cosmogenetech); lane 2, PCR product from *S. venezuelae* Hindbac tDNA; lane 3, PCR product from *S. venezuelae* tDNA.

**Figure S1.**

**
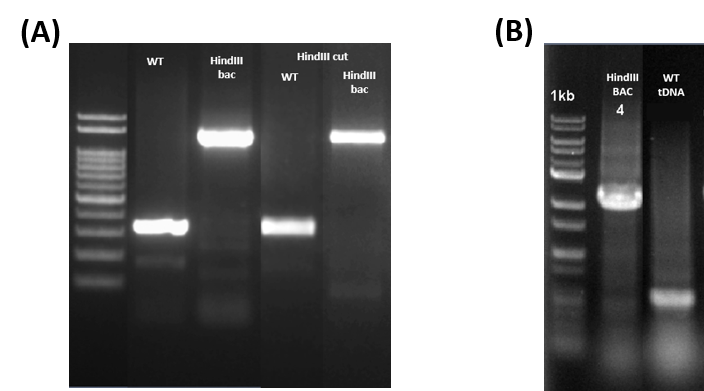
**
