# Supplementary material for: Heterologous expression of pikromycin biosynthetic gene cluster using Streptomyces artificial chromosome system
Source: Microb Cell Fact. 2017 May 31;16:96. doi: 10.1186/s12934-017-0708-7 (PMC5452415; doi:10.1186/s12934-017-0708-7)
Supplement: Supplementary file 2 — Additional file 2: Figure S2. Confirmation of pPik001 (A) PCR analysis using randomly selected primer within pikromycin biosynthetic gene cluster. Amplicons were loaded on a gel in order of pikD-kan (lane 2-3), PikAII (lane 4-5), pikRI-pikAI (lane 6-7), pikD check primers (lane 8-9). Lane 1, 1kb DNA ladder (Cosmo genetech); lane2, 4, 6, 8, S. venezuelae tDNA; lane 3,5,7,9, pPik001. (B) Enzyme mapping using various restriction enzymes. Digestion mixtures were loaded on a gel in order of BglII digestion (1-2), PstI digestion (3-4), BglII and PstI digestion (5-6). M, λ-HindIII DNA ladder; 1, 3, 5, pSBAC; 2, 4, 6, pPik001. [file 12934_2017_708_MOESM2_ESM.docx]

**Figure S2.** Confirmation of pPik001 (A) PCR analysis using randomly selectedprimer within pikromycin biosynthetic gene cluster. Amplicons were loaded on a gel in order of pikD-kan(lane 2-3), PikAII(lane 4-5), pikRI-pikAI(lane 6-7), pikD check primers(lane 8-9). Lane 1, 1kb DNA ladder (Cosmo genetech); lane2, 4, 6, 8, *S. venezuelae* tDNA; lane 3,5,7,9, pPik001. (B) Enzyme mapping using various restriction enzymes. Digestion mixtures were loaded on a gel in order of *Bgl*II digestion (1-2), *Pst*I digestion (3-4), *Bgl*II and *Pst*I digestion (5-6). M, λ-*Hind*III DNA ladder; 1, 3, 5, pSBAC; 2, 4, 6, pPik001.

**Figure S2.**

**
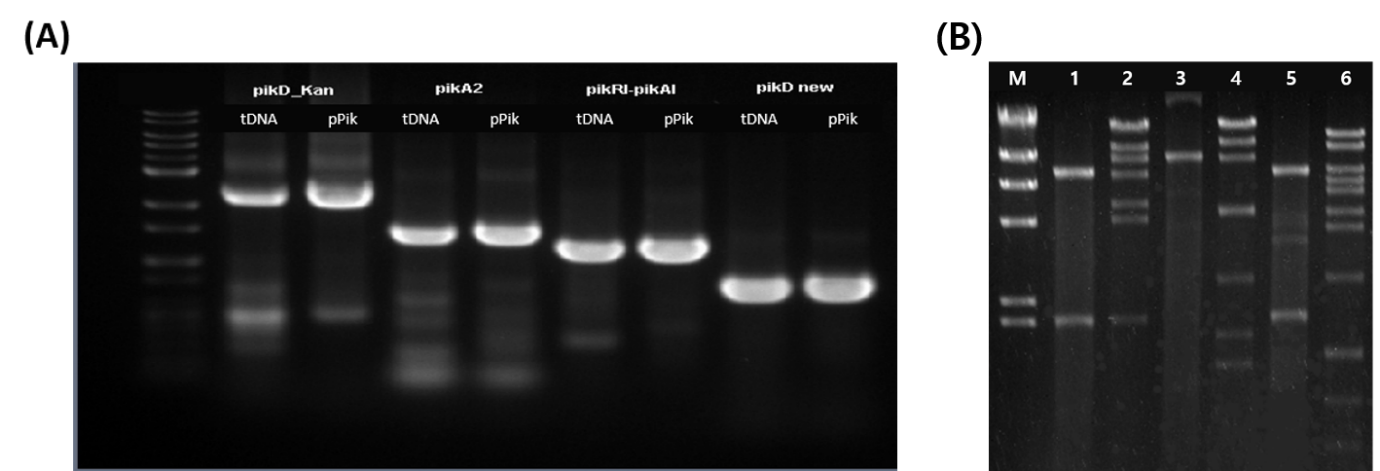
**
